# CBP/CREB Regulates the Proliferation and Apoptosis of Cardiomyocytes by Interacting With SERCA

**DOI:** 10.1111/jcmm.70426

**Published:** 2025-02-19

**Authors:** Yiran Zhouguo, Zhiyong Yuan, Mannan Abdul, Shun Xi, Tao Wei, Wei Yan, Yanan Wang, Rui Guo, Quansheng Xing, Qing Zhou

**Affiliations:** ^1^ Xuzhou Medical University Xuzhou China; ^2^ Heze Medical College Heze China; ^3^ Department of Anesthesiology, EENT Hospital of Fudan University Shanghai China; ^4^ Yangyi Education Xuzhou China; ^5^ Women & Children's Hospital Qingdao University Qingdao China; ^6^ The Affiliated Hospital of Xuzhou Medical University Xuzhou China

**Keywords:** CBP, cell proliferation, congenital heart disease, SERCA, Tetralogy of Fallot

## Abstract

Tetralogy of Fallot (TOF) is a common congenital heart disease. In this study, we proposed that cAMP response element‐binding protein (CREB)‐binding protein (CBP) regulates the proliferation and apoptosis in TOF by interacting with the sarco/endoplasmic reticulum Ca^2+^‐ATPase (SERCA). To confirm this, we collected right ventricle tissue samples from TOF patients during surgery to correct the deformity and from the donors. We performed IHC, IF, RT‐qPCR, WB and ChIP experiments. The analysis of these experiments shows that the expression of CBP is higher in TOF patients than in healthy individuals. Further, the RT‐qPCR results indicated that the CBP and SERCA mRNA in TOF patients were significantly higher than in the healthy donors. Similarly, WB results suggested that the expression of CBP and SERCA was predominantly elevated in TOF patients compared to healthy individuals. Further, the AC16 cell line with CBP knockdown reveals high expression of the Edu compared to normal cells, and the percentage of the cell cycle in the M phase was elevated in the CBPi group. In addition, the CCK‐8 cell viability assay showed more proliferation in the CBPi group than in the control group at different time points. Moreover, the RT‐qPCR results indicated a lower expression of SERCA after the knockdown of CBP and CREB. Finally, the ChIP assay shows that CREB binds to the promoter of SERCA, and the CBP enrichment decreased after the CREB knockdown. In conclusion, these results suggest that CBP interacts with SERCA to regulate cell proliferation and apoptosis during heart development and that up‐regulation of CBP leads to TOF.

## 1 Introduction

Malformations of the heart represent the most common congenital disability in humans, with an incidence of nearly 1% of all live births [1]. There is a long and remarkable history of clinical recognition, therapeutic opportunities and understanding of congenital heart disease (CHD) developmental, genetic and molecular origins [2, 3, 4]. However, the aetiology of the majority of CHDs is poorly understood. The reasons are mainly due to their complexity, as most are predicted to be multifactorial, with elaborate genetic and environmental interactions in their aetiology [5]. One severe form of CHD is Tetralogy of Fallot (TOF), representing the most common form of cyanotic CHDs [6]. TOF characterises four features: a narrowing of the right outflow tract (pulmonary stenosis), an overriding aorta, a ventricular septal defect and right ventricular hypertrophy [7, 8, 9]. Recently, the affected genes have played essential roles in apoptosis and cell growth, as well as in the sarcomere assembly and the neural crest in TOF [10, 11, 12].

Findings in animal models suggest that the cardiac neural crest influences the development of myocardial Ca2+ channels [10, 13]. Hence, a decrease in L‐type Ca2+ current was found in ventricular myocytes after cardiac neural crest ablation [14, 15]. In addition, the impaired excitation‐contraction coupling is independently caused by impaired sarcoplasmic reticulum (SR) function during embryonic dysplasia [16, 17]. In line, sarcoplasmic/endoplasmic reticulum Ca^2+^‐ATPase (SERCA) recycles calcium from the cytosol back to the endoplasmic reticulum (ER) with the assistance of ATP [18, 19]. Moreover, decreased SERCA expression and activity have been observed in cases of myocardial ischaemic reperfusion injury [20, 21]. Like endothelial function, calcium homeostasis is essential for cardiomyocytes [22]. Mild intracellular calcium overload induces cardiomyocyte spasm, whereas excessive intracellular calcium fluctuation promotes apoptosis [23]. A previous study also concluded that cAMP response element‐binding protein (CREB)‐binding protein (CBP) is involved in apoptosis in the myocardium during embryogenesis development [24].

However, the role of cardiomyocyte proliferation or apoptosis in association with CBP and SERCA needs further investigation. Therefore, we investigate the relationship between CBP and SERCA signalling pathways to regulate cardiomyocyte proliferation or TOF apoptosis.

## 2 Methods

### 2.1 Human Tissue Samples

3–5 g of the right ventricle wall biopsies of TOF patients (*n* = 7) were obtained immediately after starting cardiopulmonary bypass. Then, the cardiac tissue samples were collected from the organ donors without CHD (*n* = 7) for the control group. The tissues were snap‐frozen in liquid nitrogen and then stored at −80°C.

### 2.2 Cell Transfection

A human cardiomyocyte cell line AC16 (Zhongke quality inspection Biotechnology CO Ltd. Beijing). The culture conditions for AC16 cells are as follows: 10% fetal bovine serum and a 5% penicillin–streptomycin mixture are thoroughly mixed in the DMEM basal medium. After cell passage, the cells are placed in a cell incubator at 37°C with a carbon dioxide concentration of 5% for culture. AC16 cells were divided into two different groups, including the control group, and transfected with small interfering RNA (siRNA, sense 5′ to 3′) (GAGGUCG UUACAUAAATT antisense5′ to 3′UUUAUGUAAACGCGACCUCCT) and si‐CREB (siRNA, sense5′ to 3′) (GCCACAGAUUGCCACAUUATT antisense5′ to 3′UAAUGUGGCAAUCUGUGGCTT). The plasmids were purchased from Genepharma (Shanghai, China). Each experiment was repeated three times.

### 2.3 CCK8 Kit Analysis

Collect cells in the logarithmic growth phase, digest them with trypsin, then collect by centrifugation. Resuspend the cells in the medium and count them. Inoculate 2000 cells per well into a 96‐well plate, with 100 μL per well. Set 4 replicate wells for each group, and fill the edge wells with sterile PBS. After 0, 12, 24, 36, 48 and 72 h incubation, 10 μL CCK8 solution was added to each well and the 96‐well plates were continuously incubated at 37°C in a 2% CO_2_ incubator for 2 h. The absorbance value of each well solution was measured by a microplate reader using a 450 nm optical filter to determine AC16 cell proliferation.

### 2.4 RT‐qPCR

The tissue homogenates or cells (100 μL) were placed in the reaction tube. Repeatedly, 1 mL TRIzol (Solarbio, Beijing, China) was added using a pipette until completely dispersed. Then, it was mixed with 200 μL chloroform and incubated for 15 min at room temperature. After incubation, the homogenated tissue or cell was centrifuged at 12,000 rpm for 15 min at 4°C. The aqueous phase was carefully collected and mixed thoroughly with 0.5 mL isopropanol. The samples were centrifuged at 12,000 rpm for 10 min at 4°C after incubation for 10 min at room temperature. The supernatant was taken, and the RNA pellets were washed with 1 mL 75% ethanol and centrifuged at 8000 rpm for 5 min at 4°C. Then again, the supernatant was removed, and the RNA pellet was dried under vacuum and dissipated in 20 μL DEPC‐treated water. The concentration of RNA was measured. Then, 2 μg RNA was mixed with TaqMan reverse transcription reagent (Roche, Basel, Switzerland) and incubated at 42°C for 50 min to prepare complementary DNA (cDNA). Primers used for PCR were synthesised by Genepharma (Shanghai) (SERCA2: *ATP2A1‐1F*: GCCAGAGATGGGGAAGG 552564.164133; *ATP2A1‐1R*: GGCGAGGATTCGGATGT 684664.158; CBP: *CREBBP‐1F*: GCTGTGGACGCAAGTATG; *CREBBP‐1R*: GCCCTGGATCTCTGTGAA). Glyceraldehyde‐3‐phosphate dehydrogenase (GAPDH) was used as the reference. Each experiment was repeated three times.

### 2.5 Flow Cytometry

After 48 h transfection, cells were trypsinised (0.25%) without EDTA, collected in a flow tube, and centrifuged to remove the supernatant. Cell pellets were centrifuged to remove the supernatant. Annexin‐V/fluorescein isothiocyanate (FITC) staining buffer (Shuojia Biotech, Shanghai, China) was prepared according to the manufacturer's instructions by mixing Annexin‐V‐FITC, PI and HEPES buffer from the kit at a ratio of 1:2:50. In every 100 μL Annexin‐V/propidium iodide (PI) staining buffer, 1 × 106 cells were re‐suspended and mixed with 1 mL HEPES buffer after 15‐min incubation. Cell apoptosis was detected using a flow cytometer (BD Accouri TM C6 Plus, BD Biosciences, USA). The maximal FITC absorbance was 488 nm, and the excitation wavelength was 525 nm. The PI‐DNA complex's maximal absorbance and emission wavelength were 535 nm and 615 nm, respectively. Each experiment was repeated 3 times.

### 2.6 Western Blot

Proteins were extracted from tissues or cells using radioimmunoprecipitation assay lysis buffer and supplemented with phenylmethylsulfonyl fluoride (R001, Solarbio) precooled at 4°C. The protein concentration in each sample was determined using the Bicinchoninic acid (BCA) kit (Yeasen, Shanghai, China). Equal amounts of protein from each sample were separated by sodium dodecyl sulfate‐polyacrylamide gel electrophoresis (SDS‐PAGE) and transferred to polyvinylidene fluoride (PVDF) membranes. The membrane was blocked with 5% BSA for 1 h at room temperature and incubated with primary antibodies antirabbit overnight at 4°C. CBP (Proteintech, rabbit 22277‐1‐AP, 1:2000) and SERCA (Santa, mouse SC‐376235, 1:500) were the primary antibodies used. After incubation with primary antibodies, the membranes were washed 3 times with TBST and incubated with horseradish peroxidase (HRP) complex antirabbit immunoglobulin G (IgG 1:5000) for 1 h at room temperature. Then, they were washed three times with TBST, and the membrane was incubated with electrogenerated chemiluminescence (ECL) (LianShuo Biological, Shanghai, China) to obtain images. The results were analysed by ImageJ software. The relative protein content was represented by the grey value of the corresponding protein band and grey value of tubulin. Each experiment was repeated three times. (Western blot bands and graph of cleaved caspase 3/total caspase 3 ratios are presented in Figure S1).

### 2.7 IHC Staining

The heart tissues were fixed in 4% paraformaldehyde for 24 h. Fixed tissues were dehydrated using gradient ethanol, embedded with paraffin and sliced to 4‐μm thickness. The sections were dewaxed in xylene, rehydrated in upgraded ethanol and washed three times with distilled water. After retrieving the antigens, the sections were kept in a citrate buffer and boiled for 11 min. After cooling, the sections were washed in Tris buffer and incubated in Bovine Serum Albumin (BSA) for 10 min. After antigen retrieval, the sections were incubated in the primary antibody (mouse monoclonal antibody) CBP (1:100, Proteintech, rabbit 11149‐1‐AP) for 1 h at room temperature. The sections were washed in Tris buffer (pH 7.4) and incubated in HRP‐conjugated secondary antibody (1:100, Abcam, UK) for 1 h. They were incubated with DAB for 10 min, washed in Tris buffer (pH 7.4) and counterstained with haematoxylin, and the sections were washed under tap water, dehydrated in a graded concentration of alcohol, cleared in xylene and covered with a cover slip. The density of CBP was observed in five coronal sections with ×400 magnification using a microscope (OlympusAX70).

### 2.8 ChIP

Following the manufacturer's instructions, ChIP assays were performed with a ChIP Kit (beyotime, China). Briefly, AC16 cells were cross‐linked with 1% formaldehyde for 10 min and then quenched with glycine. Cell lysates were then sonicated to generate chromatin fragments and then immunoprecipitated with CREB antibody (Cat. #9197; CST, China), CBP antibody (Cat. #7389; CST, China) and IgG antibody (Cat. 12‐371; Millipore) was used as the negative control. The ChIP primer sequences: F: 5′‐CTCCTGGTGCGGATTCTCC‐3′; R: 5′‐GATGGCGTTCTCTGCGTTC‐3′.

### 2.9 Edu

To assess the proliferation rate of cultured cells, we employed a cell proliferation assay, specifically utilising the MTT assay method. Initially, cells were trypsinised, counted and seeded into a 96‐well plate at a density of 5000 to 10,000 cells per well. After an overnight incubation at 37°C in a 5% CO_2_ atmosphere to allow cell adherence, the cells were exposed to various experimental treatments, including control wells with no treatment or vehicle‐only treatment. Following the treatment period, 10 μL of MTT reagent was added to each well and the plate was incubated for an additional 3–4 h to facilitate the formation of formazan crystals. The culture medium was then carefully removed, and 100 μL of DMSO was added to dissolve the formazan crystals. The absorbance of each well was measured at 570 nm using a microplate reader, providing an indication of cell viability and proliferation. The relative cell proliferation was calculated by comparing the absorbance values of treated samples to those of the control samples. This method effectively allowed us to quantify the effects of various treatments on cell proliferation, providing valuable insights into the cellular response to different experimental conditions.

### 2.10 Statistical Analysis

Data were expressed as mean ± standard deviation (SD) and analysed by SPSS 21.0 software (IBM, Armonk, NY, USA), with *p* < 0.05 as a level of statistical significance. For data conforming to normal distribution and homogeneity of variance, the unpaired *t*‐test was used to compare the two groups. One‐way analysis of variance (ANOVA) was used to compare data among different groups with the post hoc Tukey's test. Repeated ANOVA measurements with post hoc Tukey's test was used to compare the data at multiple time points among different groups.

## 3 Results

### 3.1 CBP/SERCA Induces TOF in Humans

**FIGURE 1:**
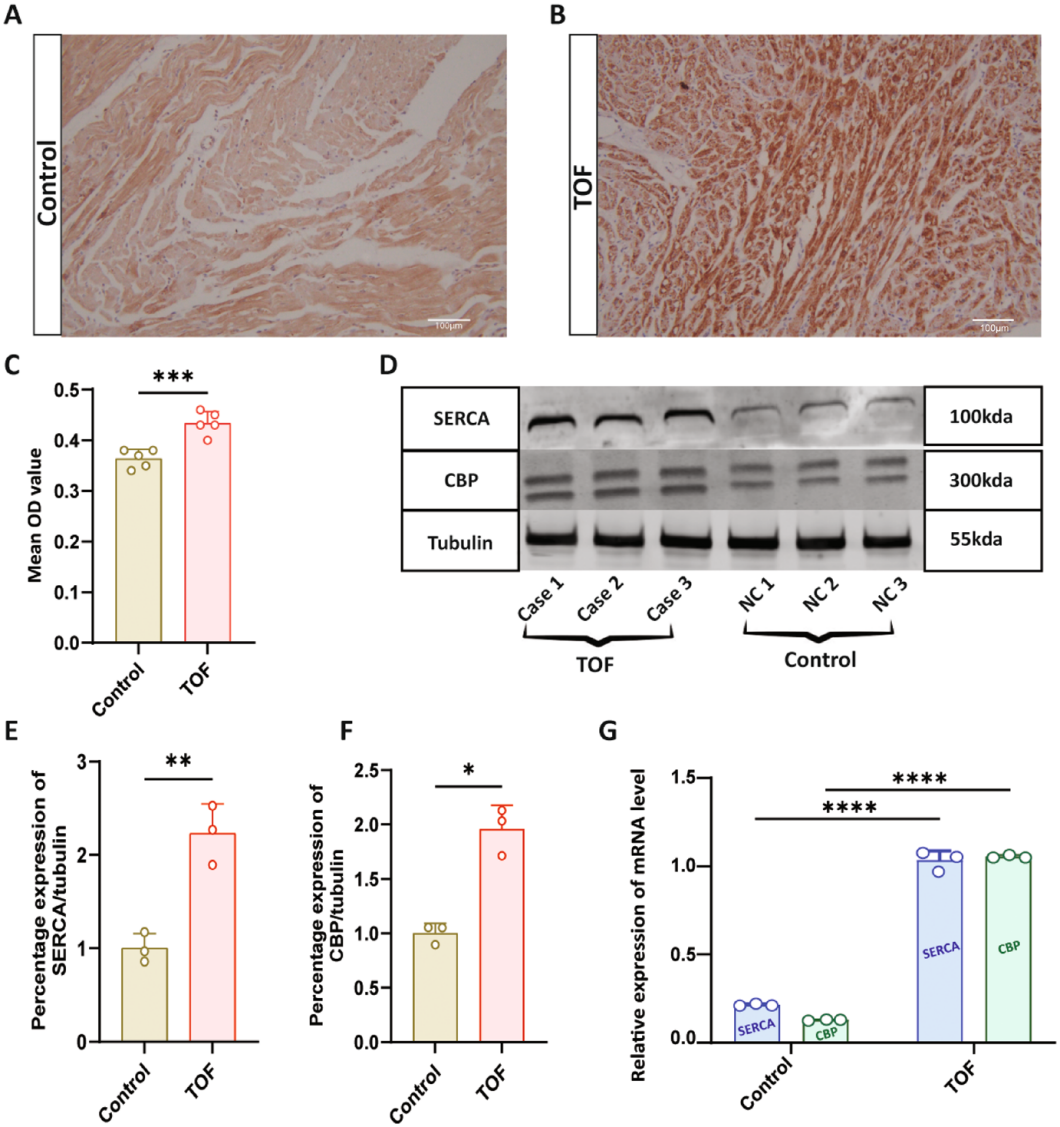
CREB‐binding protein (CBP) and sarco/endoplasmic reticulum Ca^2+^‐ATPase (SERCA) are inducing TOF pathology in humans. (A, B) IHC image of CBP in the Human Heart. (C) Representing the quantitative analysis of the IHC image, Scale bar = 100 μm, ***p < 0.001. (D) Representation of WB of the human heart sample. (E) Quantitative analysis regarding the percentage expression of SERCA\Tubulin between Control and TOF **p < 0.01. (F) Quantitative analysis regarding the percentage expression of CBP\Tubulin between Control and TOF *p < 0.05. (G) Representation of quantitative analysis of relative expression of CBP and SERCA ****p < 0.0001.

We primarily investigated the CBP and SERCA expression levels between healthy individuals (Control) and TOF patients. IHC images reveal the expression of CBP is higher in TOF patients compared to healthy individuals, and quantified results were represented in mean OD value (Figure 1A–C, *p* < 0.05). Additionally, the quantification of the WB results indicated that the expression of CBP and SERCA was predominately elevated in the TOF patients compared to the healthy individuals (Figure 1D–F, *p* < 0.05). These results suggested that the expression of the CBP and SERCA might induce TOF pathology. Further, the RT‐qPCR results indicate that mRNA in the TOF patients was significantly higher than the control at the molecular level (Figure 1G, *p* < 0.05).

### 3.2 CBP Deletion Induces Apoptosis and Can Inhibit Proliferation

**FIGURE 2:**
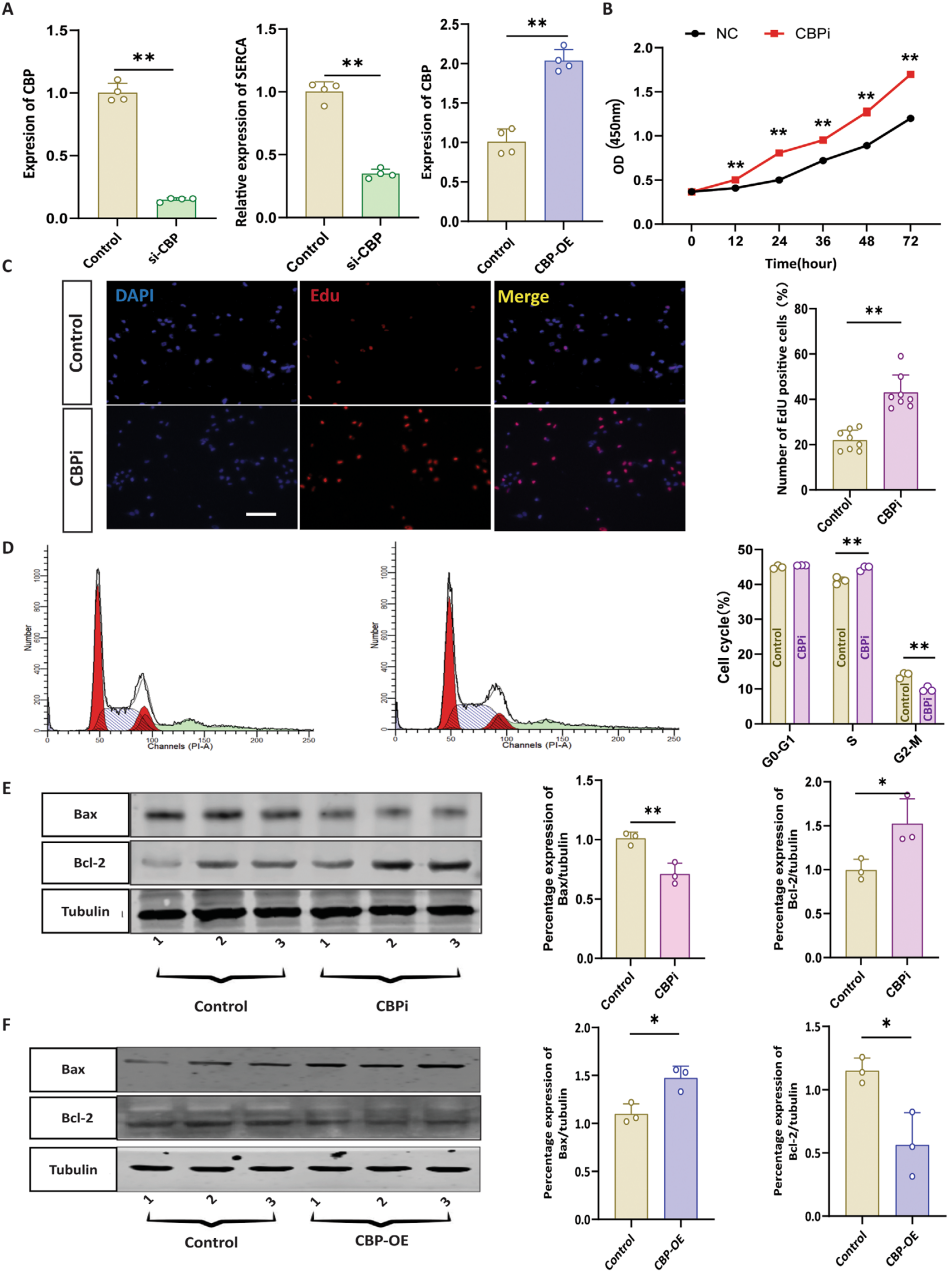
CREB‐binding protein (CBP) deletion induces apoptosis and can inhibit proliferation. (A) Representing the mRNA expression of CBP [Control and si‐CBP, **p < 0.01], relative expression of sarco/endoplasmic reticulum Ca^2+^‐ATPase (SERCA) [Control and si‐CBP, **p < 0.01], relative expression of CBP [Control and CBP‐OE, **p < 0.01] (B) Representing the cell viability analysis measured by the CCK8 assay **p < 0.01. (C) Representing IF image of the control and CBPi knockout cells and quantitative analysis of EdU positive cells per section, **p < 0.01. Scale bar = 100 μm. (D) Representing the life cycle of the control and CBPi knockout cells and quantitative analysis of the cell cycle **p < 0.01. (E) Representing the WB image of the Bax and Bcl‐2 and the quantitative analysis of the Bax and Bcl‐2 of control and CBPi. **p < 0.01, *p < 0.05. (F) Representing the WB image of the Bax and Bcl‐2 and the quantitative analysis of the Bax and Bcl‐2 of control and CBP‐OE. *p < 0.05, *p < 0.05.

Secondly, to confirm the molecular mechanism of CBP‐mediated apoptosis and its effect on inhibiting proliferation in vitro, we used a human cardiomyocyte AC16 cell line. The AC16 cells were transfected with small interfering RNA (siRNA) to knock down CBP (si‐CBP) in a controlled environment. We found that the results of the rt‐qPCR indicate that the expression of CBP in the control was significantly higher than in the si‐CBP group (Figure 2A, *p* < 0.05). Cell viability was assessed using the CCK‐8 assay, which was increased significantly after CBP knockdown (Figure 2B, *p* < 0.01). The EdU assay demonstrated a significant increase in cell proliferation after CBP knockdown (Figure 2C, *p* < 0.05), indicating that the growth rate of cells was gradually accelerated after knocking down CPB. A flow cytometry study was conducted to determine whether the effects of CBPi were cell cycle‐dependent. After exposing the cells to siRNA, the number of arrests in each cycle phase (including the G1, G2 and S phases) was measured. After the treatment with siRNA, the percentage of the cells in the G2/M phase was elevated in the CBPi group; (Figure 2D, *p* < 0.05). Flow cytometry was performed to determine if the growth‐inhibitory effects of CBP knockdown were cell cycle‐dependent. After siRNA exposure, the number of cells arrested in each cell cycle phase (G1, G2 and S phases) was measured. siRNA treatment increased the percentage of cells in the G2/M phase in the CBP knockdown group (Figure 2D, *p* < 0.05). Furthermore, Western blot analysis indicated that Bax protein expression decreased in the CBP knockdown group compared to the control group (Figure 2E, *p* < 0.05), while Bcl‐2 protein expression increased in the CBP knockdown group (Figure 2E, *p* < 0.05). These results suggest that CBP regulates both cell proliferation and apoptosis in AC16 cells.

### 3.3 CBP/CREB Complex Regulates the Transcription of SERCA

**FIGURE 3:**
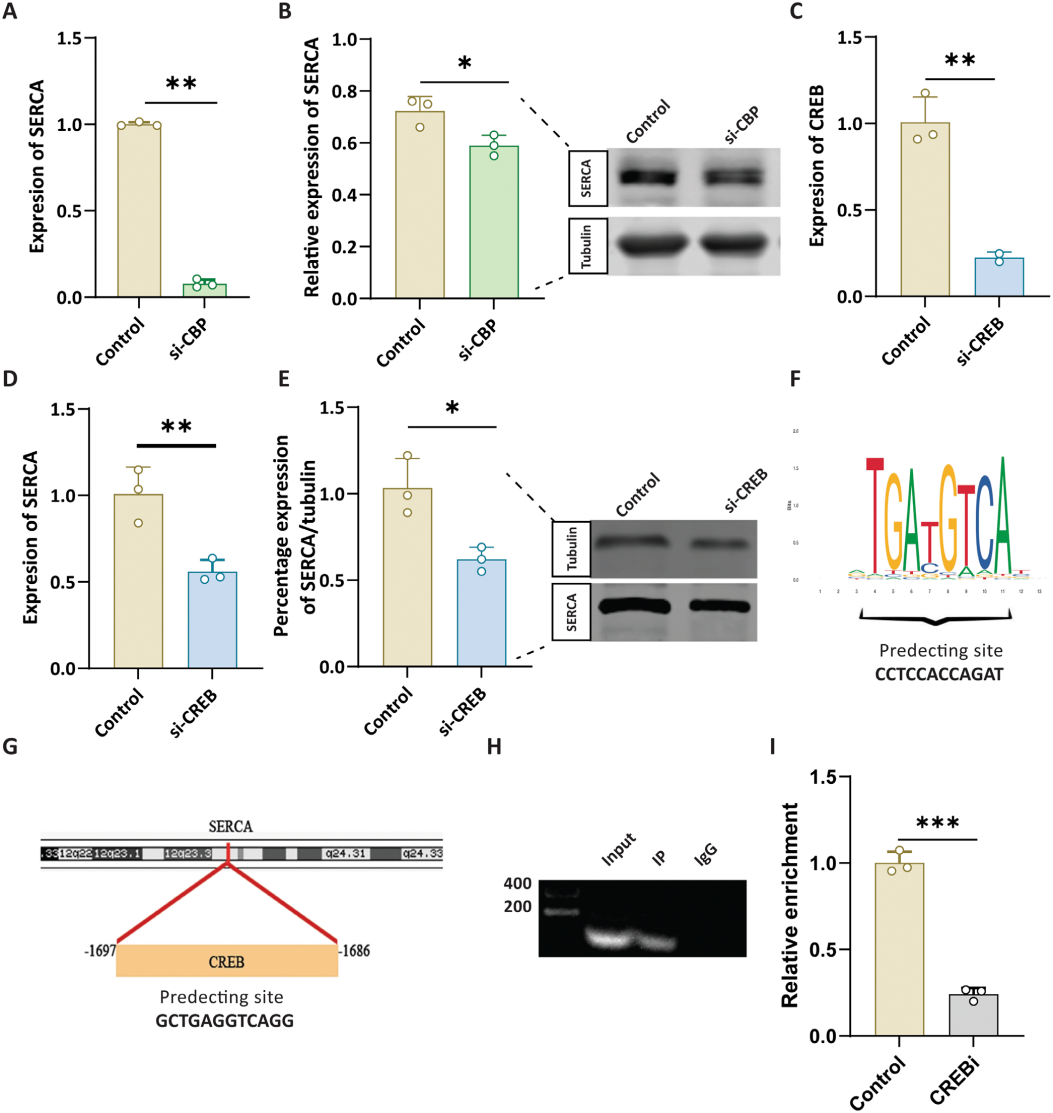
ChiP and Co‐immunoprecipitation (IP) showing the interaction between the CREB‐binding protein (CBP) and sarco/endoplasmic reticulum Ca^2+^‐ATPase (SERCA). (A) Representing the RT‐qPCR quantitative analysis of the SERCA **p < 0.01. (B) Representing the WB image of SERCA and quantitative analysis of the SERCA, *p < 0.05. (C) The RT‐qPCR quantitative analysis of the CERB after the knockdown of CREB **p < 0.01 (D) represents the RT‐qPCR quantitative analysis of the SERCA **p < 0.01. (E) Representing the WB image of SERCA and quantitative analysis of the SERCA in si‐CREB *p < 0.05. (F) Representing the potential cofactor of the genome. (G) Representing the genome location on the chromosome (H) representing CO‐IP expression of CBP and SERCA. (I) representing the relative enrichment of the genome expression ***p < 0.001.

We first used RT‐qPCR and WB to detect the expression of SERCA after CBP knockdown and found that SERCA was downregulated after CBP knockdown(Figure 3A,B, *p* < 0.05)We observed that SERCA expression decreased significantly after CREB knockdown, similar to the reduction seen after CBP knockdown (Figure 3C–E, *p* < 0.05). Next, we nominated the binding sites of CREB to *SERCA* promoters on JASPAR and selected the site with the highest score for the study (Figure 3F,G, *p* < 0.05). We found that CREB and SERCA promoters were bound by chip (Figure 3H), and the enrichment of CBP and SERCA promoters decreased after CREB was knocked down (Figure 3I, *p* < 0.01). These results suggest that the CBP/CREB complex regulates SERCA transcription.

## 4 Discussion

Extensive scientific literature indicates that Ca2+ dysregulation in cardiomyocytes is crucial to the underlying cardiac dysfunction [25]. Our study showed that the expression of CBP is higher in TOF patients than in healthy individuals. Further, the PCR results indicate that the CBP and SERCA mRNA in the TOF patients were significantly higher than the control at the molecular level. Additionally, the quantification of the WB results indicated that the expression of CPB and SERCA was predominately elevated in the TOF patients, which corresponded to healthy individuals. However, a comparative study in children with congenital heart defects showed no difference in SERC2a expression levels [16]. Nevertheless, others have demonstrated that CBP triggers apoptosis through the transcriptional induction of the pro‐apoptotic Bcl‐2 in the heart [24]. Therefore, these results suggest that the expression of the CBP and SERCA might be the inducing factor for the TOF pathology. Further, CBP‐mediated proliferation in vitro showed that the CBPi cells express higher Edu levels than the control group. In addition, we also found that the CCK‐8 cell viability assay results showed the stimulation of proliferation in the CBPi group compared with the control group at different time points. These results suggest that the knockdown of the CBP increased cell proliferation. A previous study indicated that CBP plays a vital role in tumour‐suppressing activity [26]. Furthermore, others reported that CBP enhances the activity of the p53 gene via endogenous activation of the p21, resulting in the suppression of cell proliferation and apoptosis [27]. Additionally, a study has shown that p53 directly binds to the SERCA at the endoplasmic reticulum, leading to Ca^+2^ overloading and apoptosis [28]. In our study, RT‐qPCR analysis revealed a significant reduction in the expression of CBP and SERCA following siRNA transfection compared to the control group. This suggests a pivotal role of CBP and SERCA in regulating cell proliferation and apoptosis during cardiac embryonic development. CBP, known as a powerful transcriptional coactivator, influences the transcription of a wide array of genes. Our findings indicated that knocking down CBP led to a decrease in SERCA expression, suggesting that CBP may play a regulatory role in SERCA expression. Given the well‐documented interactions between CREB and CBP, we further examined the effect of CREB on SERCA expression and observed a similar decrease in SERCA levels. Additionally, our analysis demonstrated that CREB could directly bind to SERCA promoters, thereby regulating its transcription. This was supported by the significant binding observed between CREB and SERCA promoters. The CREB–CBP complex's ability to bind to SERCA promoters suggests a coordinated regulation of SERCA transcription, resulting in its high expression. These results indicate that during cardiac embryonic development, CBP regulates cell proliferation and apoptosis through the modulation of SERCA expression. Furthermore, upregulation of CBP has been associated with CHDs, including TOF. Our study found that CBP and SERCA expression levels were significantly elevated in TOF patients compared to healthy controls, indicating their potential role in TOF pathology. Knocking down CBP in AC16 cells led to decreased cell viability, increased cell proliferation, and altered cell cycle and apoptosis‐related protein expressions. Additionally, both CBP and CREB knockdowns resulted in reduced SERCA expression, highlighting their regulatory role in SERCA transcription. These findings suggest that the CBP/CREB complex plays a crucial role in regulating SERCA and may contribute to the pathology of TOF. Future studies are essential to explore the detailed mechanisms by which CBP regulates SERCA during embryonic development, which could provide insights into potential therapeutic targets for CHDs.

## Author Contributions

**Yiran Zhouguo:** formal analysis (equal), investigation (equal), methodology (equal), writing – original draft (equal). **Zhiyong Yuan:** project administration (equal), supervision (equal). **Mannan Abdul:** writing – original draft (equal), writing – review and editing (equal). **Shun Xi:** formal analysis (equal), investigation (equal), methodology (equal). **Tao Wei:** data curation (equal), formal analysis (equal). **Wei Yan:** investigation (equal), resources (equal). **Yanan Wang:** writing – review and editing (equal). **Rui Guo:** conceptualization (equal), supervision (equal), writing – original draft (equal), writing – review and editing (equal). **Quansheng Xing:** conceptualization (equal), funding acquisition (equal), supervision (equal), writing – review and editing (equal). **Qing Zhou:** formal analysis (equal), methodology (equal), writing – original draft (equal).

## Ethics Statement

All procedures were completed under the regulations of the ethical committee of the Women & Children's Hospital, Qingdao University, [QFELLY‐Y‐2020‐63].

## Consent

Written informed consent was obtained from the parents.

## Conflicts of Interest

The authors declare no conflicts of interest.

## Supporting information


**Figure S1** Western blot bands and data of capase‐3/cleaved capase‐3/tubulin (A) Representing WB image of capase‐3/cleaved capase‐3/tubulin (CBPi) and quantitative analysis of NC and CBPi. ****p < 0.01. (B) Representing WB image of capase‐3/cleaved capase‐3/tubulin (CBP‐OE) and quantitative analysis of NC and CBP‐OE ***p < 0.01.

## Data Availability

The data that support the findings of this study are available from the corresponding author upon reasonable request.
