# Supplementary figures and images for: CBP/CREB Regulates the Proliferation and Apoptosis of Cardiomyocytes by Interacting With SERCA
Source: J Cell Mol Med. 2025 Feb 19;29(4):e70426. doi: 10.1111/jcmm.70426 (PMC11837278; doi:10.1111/jcmm.70426)

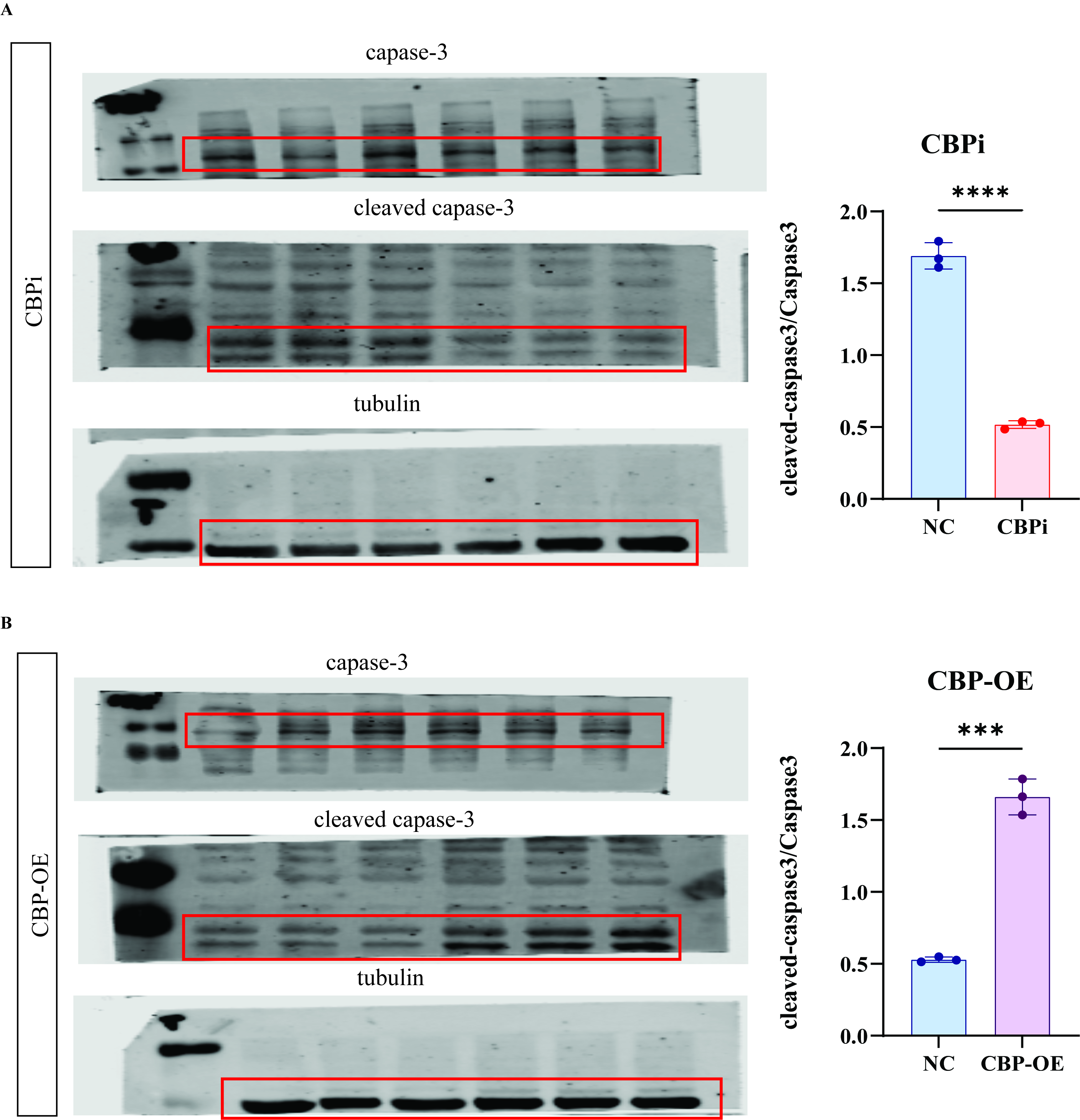

Supplement: Supplementary file 1 — Figure S1 Western blot bands and data of capase‐3/cleaved capase‐3/tubulin (A) Representing WB image of capase‐3/cleaved capase‐3/tubulin (CBPi) and quantitative analysis of NC and CBPi. ****p < 0.01. (B) Representing WB image of capase‐3/cleaved capase‐3/tubulin (CBP‐OE) and quantitative analysis of NC and CBP‐OE ***p < 0.01. [file JCMM-29-e70426-s001.tif]
